# 2297 The direct effect of trimethylamine *N*-oxide (TMAO) on cardiac muscle contractile mechanics

**DOI:** 10.1017/cts.2018.130

**Published:** 2018-11-21

**Authors:** Carlee I. Oakley, David Sanborn, Nikita Rafie, Matt Hendrix, Michael Grillo, Julian Vallejo, Jason R. Stubbs, Tilitha Shawgo, Emmanuel Daon, George Zorn, Michael J. Wacker

**Affiliations:** 1 University of Kansas Frontiers; 2 Cardiovascular Research Institute, University of Kansas Medical Center; 3 University of Missouri-Kansas City School of Medicine

## Abstract

OBJECTIVES/SPECIFIC AIMS: The objective of this study was to determine if trimethylamine *N*-oxide (TMAO) alone could acutely alter cardiac contractile function on a beat-to-beat basis. METHODS/STUDY POPULATION: CD1 adult mouse hearts were extracted, attached to a force transducer, oxygenated, and paced within an organ bath. Changes in contractility were measured after pipetting or reverse perfusing TMAO through the aorta via a modified Langendorff apparatus to facilitate TMAO delivery into the myocardium. To determine if our findings translated to the human heart, we performed contractility experiments using human right atrial appendage biopsy tissue retrieved during cardiopulmonary bypass procedures. To investigate whether TMAO alters contractile rate, in a separate series of experiments, the atria and sinoatrial node of isolated hearts were kept intact to allow for spontaneous beating without artificial pacing and were treated with TMAO or vehicle. In addition, intracellular calcium measurements were performed on spontaneously beating embryonic rat cardiomyocytes after TMAO or vehicle treatment. RESULTS/ANTICIPATED RESULTS: We found acute exposure to TMAO, diluted into the organ bath, increased average contraction amplitude 20% and 41% at 300 µM and 3000 µM, respectively (*p*<0.05, n=6). Langendorff reverse perfusion of mouse hearts ex vivo with 300 µM TMAO generated an even greater response than nonperfusion peripheral exposure and increased isometric force 32% (*p*<0.05, n=3). Consistent with what we observed in mouse hearts, incubation of human atrial muscle tissue with TMAO at 3000 µM increased isometric tension 31% compared with vehicle (*p*<0.05, n=4). TMAO treatment (3000 µM) also increased average beating frequency of ex vivo mouse hearts by 27% compared with vehicle (*p*<0.05, n=3) and increased the spontaneous beating frequency of primary rat cardiomyocytes by 13% compared with vehicle treatment (*p*<0.05, n=4). DISCUSSION/SIGNIFICANCE OF IMPACT: TMAO, at pathological concentrations, directly increases the force and rate of cardiac contractility. Initially, these inotropic and chronotropic effects may be adaptive during CKD; however, chronic increases in isometric tension and beating frequency can promote cardiac remodeling and heart failure. Further translational studies are needed to understand the intricate relationship between the microbiome, kidneys, and heart and to examine if TMAO represents a therapeutic target for reducing cardiovascular mortality in CKD patients.

